# Drug Molecular Immobilization and Photofunctionalization of Calcium Phosphates for Exploring Theranostic Functions

**DOI:** 10.3390/molecules27185916

**Published:** 2022-09-12

**Authors:** Iori Yamada, Kota Shiba, Tania Guadalupe Peñaflor Galindo, Motohiro Tagaya

**Affiliations:** 1 Department of Materials Science and Technology, Nagaoka University of Technology, Kamitomioka 1603-1, Nagaoka 940-2188, Niigata, Japan; 2 Center for Functional Sensor & Actuator (CFSN), Research Center for Functional Materials, National Institute for Materials Science (NIMS), 1-1 Namiki, Tsukuba 305-0044, Ibaraki, Japan; 3 General Department, National Institute of Technology, Nagaoka College, 888 Nishikatagai, Nagaoka 940-8532, Niigata, Japan

**Keywords:** theranostics, octacalcium phosphate, hydroxyapatite, inorganic–organic hybrid, photofunctionalization, europium(III), methylene blue

## Abstract

Theranostics (bifunction of therapeutics and diagnostics) has attracted increasing attention due to its efficiency that can reduce the physical and financial burden on patients. One of the promising materials for theranostics is calcium phosphate (CP) and it is biocompatible and can be functionalized not only with drug molecules but also with rare earth ions to show photoluminescence that is necessary for the diagnostic purpose. Such the CP-based hybrids are formed in vivo by interacting between functional groups of organic molecules and inorganic ions. It is of great importance to elucidate the interaction of CP with the photofunctional species and the drug molecules to clarify the relationship between the existing state and function. Well-designed photofunctional CPs will contribute to biomedical fields as highly-functional ormultifunctional theranostic materials at the nanoscales. In this review, we describe the hybridization between CPs and heterogeneous species, mainly focusing on europium(III) ion and methylene blue molecule as the representative photofunctional species for theranostics applications.

## 1. Introduction

The term ‘theranostics’ is a combination of therapeutics and diagnostics, which means a concept that tries to achieve therapy and diagnosis at the same time with one system [1]. Diagnosis and therapy have been performed separately using different materials and methods so far. There are various modalities of theranostics, including selective treatment through visualization of lesion sites, simultaneous treatment and diagnosis, and visualization of treatment effects. Theranostics can be achieved by designing a material that has specific accumulation into the abnormal cells (e.g., cancer cells) and has both diagnostic and therapeutic properties.

The diagnostic methods for theranostics include magnetic resonance imaging, positron emission tomography, single photon emission computed tomography, endoscopy and in vivo optical imaging. The therapeutic methods for theranostics include photodynamic therapy (PDT), gene therapy, radiotherapy, hyperthermia, photothermal therapy and chemotherapy. Among these diagnostic approaches, fluorescence endoscopy is one of the convenient and versatile diagnostic techniques; it is based on luminescence and coloration from an affected part due to the accumulation of luminescent species or dyes. For therapeutic purposes, chemotherapy and PDT using reactive oxygen species (ROS) generated by a photosensitizer are widely used because the former is applicable to various types of cancers and acts on cancers throughout the body, and the latter is non-invasive and has far fewer side effects than other techniques. These diagnostic and therapeutic methods need to work with the same base material for theranostic applications. Various materials, including polymer particles [2,3,4,5], liposomes [6,7], micelles [8], quantum dots [9,10] and bioceramic nanoparticles [11,12], have been investigated to see whether they could be used as the base material [13]. Moreover, various hybrid nanomaterials have also been developed and proposed for theranostics, combining these materials with drug molecules and imaging spices [14,15,16,17]. 

The luminescent materials that have been used in cellular imaging and bioimaging for diagnosis can be roughly categorized into the following two groups: organic molecular systems, such as fluorescent dyes, and inorganic systems, such as quantum dots and luminescent particles doped with rare earth ions. The conventional fluorescent dyes suffer from low brightness, sensitivity and quantifiability, as well as fading, which makes long-term observation difficult, leading to a narrow application range. Attempts have been made to improve the optical properties by modifying the dye molecules themselves [18,19] or by hybridizing organic dyes with various inorganic and organic materials [11,20] while ensuring safety. As a result, dyes have been designed to be excited at wavelengths that can be transmitted through biological tissue [21,22,23], and fluorescence quantum efficiency has been improved [24,25]. Nevertheless, the following challenges still remain: low water solubility, aggregate formation and inability to target cancer tissues [26]. As for inorganic systems, the quantum dots have high luminescence efficiency, brightness and light resistance [9,27,28]. However, they are composed of metallic elements, such as arsenic and cadmium, which are highly toxic and require high excitation energy, resulting in damage to normal cells [9]. These problems are essential and must be solved before testing on a living body. Doping of rare earth ions is a possible solution to the problems; these ions are highly light resistant, can be excited by visible light, emit visible light, whose bandwidth is narrow, and are non-toxic [29], leading to a promising photofunctionalization method.

**Figure 1:**
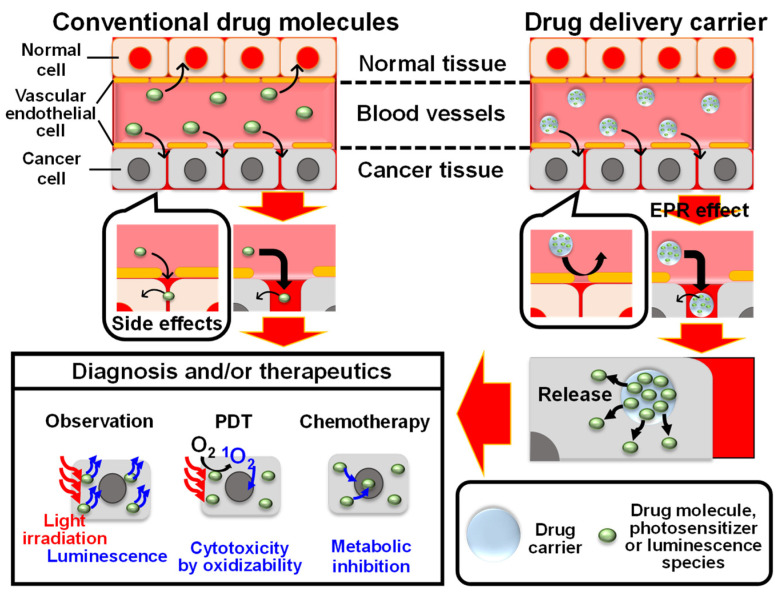
Schematic illustration of the diagnosis with luminescence and therapeutics with chemotherapy and PDT by conventional drug molecules and drug delivery carrier.

A schematic illustrates therapeutics with chemotherapy and PDT and diagnosis with luminescence by conventional drug molecules and drug delivery carriers, as shown in Figure 1. PDT kills cancer cells by ROS produced by irradiating the photosensitizer with excitation light, and it has the advantage of causing far fewer side effects than other techniques, and it can be used in combination with other therapeutic methods [13,30]. The ability of a photosensitizer to generate the singlet oxygen (^1^O_2_) is important in PDT because the ^1^O_2_ has a high ability to kill cancer cells among the ROS [31,32]. The typical photosensitizers used in PDT applications are mainly rose bengal, porphyrin analogs and methylene blue (MB^+^) [33,34,35,36]. As a side effect of the photosensitizer, photosensitivity (inflammation of the area exposed to the light) is caused by an increase in the concentration of the photosensitizer in the body due to the low accumulation of the photosensitizer in the cancer. Therefore, the patient should avoid light exposure before and after the PDT procedure. In addition, the photosensitizer loses the ^1^O_2_ generation ability due to its aggregation and degradation reaction in the cells [37]. It is important to maintain the photosensitizer molecular state that is capable of performing photofunctions and also to improve the stability of the photosensitizer and accumulation into cancer cells in PDT. In chemotherapy, anticancer drugs that cause damage to cells or inhibit cell growth act on abnormal cells, leading to cancer therapeutics. However, anticancer drugs also act on normal cells, resulting in side effects. One of the promising solutions to this problem is drug delivery; it is expected to ensure that the proper amount of anticancer drug acts only on the affected area. To achieve drug delivery, firstly, the drug accumulation in the cancer tissue must be sufficiently high [38]. The property of the cancer tissue used for this purpose is the enhanced permeability and retention (EPR) effect, which is due to the defective structure of blood vessels around cancer tissues and the lack of a lymphatic system [39]. As a result, the nanoparticles are more likely to be accumulated in the cancer tissue and are less likely to be expelled from the cancer cells compared to normal cells. Namely, if the drug molecules can be nanoparticulated or hybridized with nanoparticles, the drug molecules can be accumulated into cancer tissues. Such nanoparticles that deliver drug molecules to tissues are called drug carriers. The drug carrier materials must have high biosafety and drug loading capacity [40]. The use of drug carriers not only enhances the accumulation of drug molecules but also controls the sustained release of drug molecules by utilizing the carrier properties. Furthermore, the drug molecules supported on the drug carrier could be stabilized by interaction with carrier materials. These properties would realize the maintenance of drug molecular concentrations in the long term, which produces the medicinal effect under certain conditions (e.g., lower pH value in the cancer cell than in the normal cell [41]) and is triggered by external stimulation (e.g., heat [42], light irradiation [43], etc.).

Among the various materials for drug carriers, bioceramic nanoparticles have a guaranteed safety profile and show a variety of functions. In particular, calcium phosphate (CP) nanoparticles, which show particularly high biosafety, have an excellent adsorption capacity for poorly water-soluble anticancer drugs, and these drug molecules can be released slowly with the dissolution of CPs [12,44,45,46]. From the above point of view, CP nanoparticles are a promising base material for the realization of theranostics. However, pristine CP nanoparticles need to be functionalized properly toward theranostic applications. For this purpose, it is important to mimic the interactions between CPs and heterogeneous substances from naturally occurring hybrid nanomaterials in vivo, which are generated via mineralization processes. The mineralization process involves the uptake of heterogeneous ions from body fluids and the formation of a hybrid surface with organic molecules. This property of producing hybrids with heterogeneous substances can be used to hybridize the functional substances with the CP crystals or surfaces to provide the novel functions. In this review, we describe the basic properties of CPs, the hybrid materials of CPs with heterogeneous molecules and ions, the photofunctionalization of the CPs in addition to the hybridization with drug molecules and the photosensitizers to resultantly design the biosafety hybrid nanomaterials based on the biomineralization process.

## 2. Photofunctionalization of Calcium Phosphates Based on Biomimetic Surface Engineering

### 2.1. Octacalcium Phosphate (OCP)

#### 2.1.1. Features

**Figure 2:**
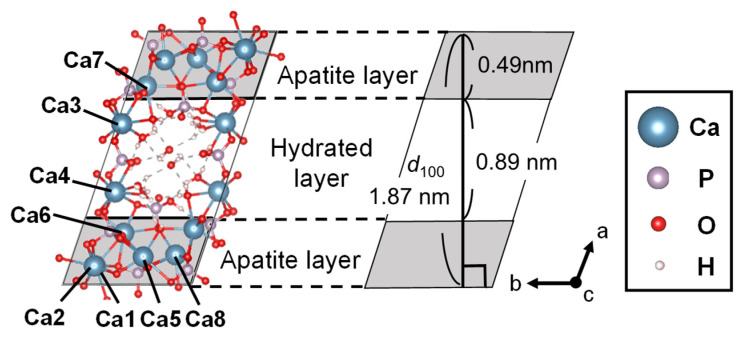
Crystal structure of OCP and distance in OCP structure indicated by *d*_100_.

Octacalcium phosphate (OCP, Ca_8_(HPO_4_)_2_(PO_4_)_4_·5H_2_O) is a type of CP and has a Ca/P molar ratio of 1.33, density of 2.61 g cm^−3^ and belongs to the triclinic crystal system and space group of Pī. The lattice constants of OCP are *a* = 19.692, *b* = 9.523, *c* = 6.835 Å, *α* = 90.15, *β* = 92.54, *γ* = 108.65° (Figure 2) [47]. The crystalline OCP consists of a characteristic layered structure with an alternating apatite layer (chemical composition: 2[Ca_3_(PO_4_)_2_∙0.5H_2_O]), which is similar to hydroxyapatite (HA), as described below, and a hydrated layer containing water molecules (chemical composition: 2[CaHPO_4_∙2H_2_O]) [48,49], which constitutes a nanospace that can contain organic molecules. OCP has eight Ca sites with different coordination environments. The solubility (−logKsp) at 25 °C and 37 °C is 96.6 and 95.9, respectively. OCP is stable in aqueous solutions at 25 °C at a pH value between 5.5 and 7.0 [50,51]. OCP is converted to HA by hydrolysis reaction. Because HA is the main inorganic component of biological bone, OCP, which is converted to HA and has a high biosafety, is used as a bone filling material [52,53].

#### 2.1.2. In Vivo and In Vitro Formation Processes

**Figure 3:**
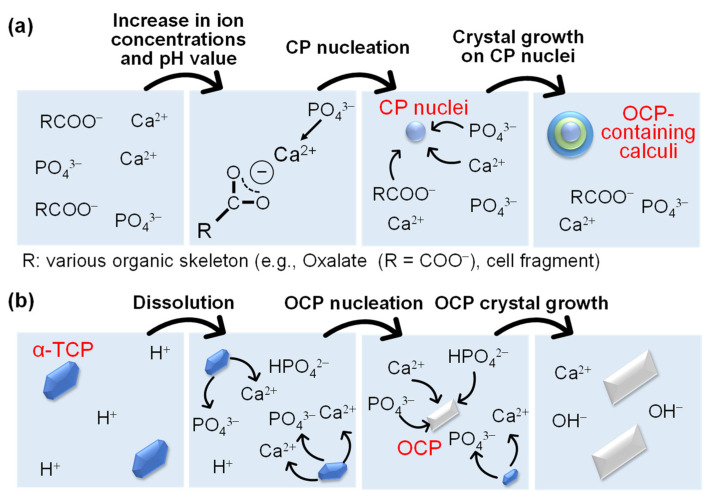
Possible illustrations of (**a**) formation process of OCP-containing calculi in vivo and (**b**) OCP synthesis by α-TCP hydrolysis in vitro.

OCP converts to HA, which is the main inorganic component of bone, in implantation experiments in vivo, suggesting that OCP would be present as a precursor of HA during the early stage of bone formation [54,55]. The formation of OCP as a precursor of tooth enamel has also been proposed, since ribbon-like crystals similar to OCP crystals are produced in the early stages of enamel formation [56,57,58]. This result suggests that OCP is formed by interacting with a protein (e.g., amelogenin) present in the early stages of enamel formation [58,59]. However, the detail of the interaction between protein and OCP crystallization has not been completely elucidated, and OCP is not clearly observed in the in vivo mineralization [60,61]. OCP is found in calculus caused by pathological calcification along with the other CPs [60,62], which means that OCP does not exist in a single phase. Although the mechanism of calculus formation is not entirely clarified, the surface of the organic matter (e.g., tissue surface and organic matter dissolved in body fluids) is assumed to be the nucleation site of the calculus precursor because the calculus often contains large amounts of organic molecules; the nucleation site is formed under a higher pH value than normal value to induce supersaturation of both Ca^2+^ ion and uric acid in body fluid. The precursor acts as a more favorable nucleation site for the new calculus layer (Figure 3a) [63,64,65]. Accordingly, the pH value, ion concentration and nucleation site are significant factors for the formation of OCP in vivo.

When we consider an in vitro process for the synthesis of OCP instead of an in vivo one, there are two main methods: a liquid–liquid process of reacting Ca and phosphate ion aqueous solutions and a solid–liquid process of other CPs hydrolysis in the aqueous solution containing phosphate ions. On the basis of these two processes, organic molecules would be added to the liquid phase to mimic the in vivo environment, control the morphology and functionalize. Compared with the other CPs, the experimental conditions are limited for OCP production. The typical efficient synthetic methods are described below.

In the liquid–liquid synthesis method with the reaction formula in Equation (1), water-soluble calcium salts (e.g., acetate (CH_3_COO^−^) [66,67,68,69,70] and nitrate (NO_3_^−^) [71]) solution is used as the Ca source and the solution of hydrogen and/or dihydrogen phosphate salts as the phosphate source (NaH_2_PO_4_ [66,67,68,69,71], Na_2_HPO_4_ [60,69], mixture of (NH_4_)_2_HPO_4_ and NH_4_H_2_PO_4_ [59,70]). Then, the Ca source solution is added to the phosphate source solution while heating above 50 °C and stirring. For the biomimetic synthesis, calcium chloride solution [60] and ammonium phosphate salts [59] can be used to synthesize the OCP at 37 °C. The OCP synthesized by dropwise addition of the Ca(CH_3_COO)_2_ aqueous solution into the NaH_2_PO_4_ aqueous solution at a pH value of 5–6 and at 70 °C is in the form of several µm sized plate-like crystals [66,67]. The OCP nanobelt crystal of one submillimeter in length and several micrometers in width is obtained by dropping the Ca(CH_3_COO)_2_ aqueous solution into the NaH_2_PO_4_ and Na_2_HPO_4_ mixture aqueous solution (pH = 5.0) in which a cetyltrimethylammonium bromide is dissolved and reacting at 60 °C for 1 h [68].

8Ca^2+^ + 2HPO_4_^2−^ + 4PO_4_^3−^ + 5H_2_O → Ca_8_H_2_(PO_4_)_6_·5H_2_O (1)

In the solid–liquid synthesis method, there are many derived methods for OCP synthesis. One of those methods is the hydrolysis of CPs (e.g., alpha-phase tricalcium phosphate (α-TCP), CaHPO_4_·2H_2_O (DCPD)) in a weakly acidic aqueous solution (e.g., 3 < pH < 7) while heating (e.g., 40–70 °C) and stirring for several hours [72,73]. In the synthesis of OCP by α-TCP hydrolysis with a dissolution reaction (Figure 3b, Equation (2)), OCP is produced in the range of the combination of lower temperature–higher pH or higher temperature–lower pH conditions. DCPD is produced at lower temperatures and/or a lower pH value, and HA is produced at higher temperatures and/or a higher pH value than these conditions [72]. The hydrolysis of α-TCP produces an aggregate of micrometer-sized strip-like OCP crystals.

3Ca_3_(PO_4_)_2_ + 7H_2_O → Ca_8_H_2_(PO_4_)_6_·5H_2_O + Ca(OH)_2_ (2)

Another synthetic method is the hydrolysis of DCPD in phosphate source solutions during heating. Specifically, DCPD is added to a (NH_4_)_2_HPO_4_ aqueous solution and stirred at 60 °C for 24 h [74]. Other methods include the reaction of calcium carbonate with phosphoric acid (Equation (3a–c)) during heating at 60 °C for 6 h [75,76]. In the case of the synthesis by adding the DCPD into the NH_4_H_2_PO_4_ aqueous solution and reacting at a pH value of 8.0 for 1 day at 60 °C, the plate and ribbon-like OCP crystals with a size of approximately 1 μm are produced [74]. On the basis of these synthetic methods, a wide variety of functionalized OCP can be synthesized with the coexistence of the substances to be hybridized in an aqueous solution.

CaCO_3_·+ 2H_3_O^+^ + → Ca^2+^·3H_2_O + CO_2_ (3a)

Ca^2+^·+ HPO_4_^2−^ + 2H_2_O → CaHPO_4_·2H_2_O (3b)

6CaHPO_4_·2H_2_O + 2Ca^2+^ → Ca_8_H_2_(PO_4_)_6_·5H_2_O +3H_2_O + 4H_3_O^+^ (3c)

#### 2.1.3. Hybridization with Organic Molecules in the Formation Processes

**Figure 4:**
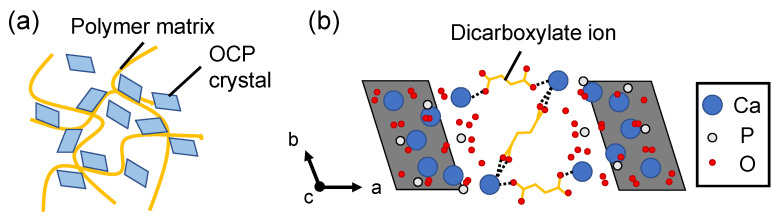
Illustration of hybrid states of (**a**) OCP/polymer complex to improve the shaping and enhancing bone regeneration and (**b**) OCPC to provide the selective adsorption and catalysis.

OCP has been used as a bone filling material, as described above. However, it suffers from poor formability, handling and strength. To improve these properties, the hybridization of OCP and polymer at the extralaminar level is carried out through imitation of the bone, which is a hybrid of collagen and HA (Figure 4a). A hybrid of OCP and collagen (OCP/Col) has been synthesized to promote bone regeneration and improve biodegradability when implanted in bone defects. A comparison of the bone formation amount with OCP alone, collagen alone and OCP/Col showed that OCP/Col had the highest bone formation amount, indicating that OCP/Col had a high osteogenic potential. The hybrid of OCP and collagen improved not only the handling performance and strength but also the osteogenic potential [53]. Gelatine is a random coil protein derived from denatured collagen and has been investigated as a matrix for bone filling materials. The hybrid of OCP and gelatine was able to efficiently repair the defect when implanted into the bone defect, and most of the hybrid disappeared after incubation, indicating that the hybrid had a high osteogenic potential and biodegradability [77]. These are extralaminar hybrids of OCP and polymer, which do not use the unique properties of the interlayer function of OCP. These hybrids are formed by interacting with polymer chains at the surface of submicron-sized OCP, and their application is limited to bone filling materials. Accordingly, the hybridization of organic molecules and OCP at the nanoscale to functionalize OCP is expected for new applications.

The hydrogen phosphate ions in the hydrated layer of OCP can be replaced with dicarboxylate ions by coexisting with dicarboxylate ions (^−^OOC–R–COO^−^) during OCP formation (Figure 4b) [78]. The substitution of linear, various side chain, unsaturated and aromatic dicarboxylic acid ions has been attempted to design novel OCP/dicarboxylate ion hybrids with a layered nanostructure for adsorbent and catalytic applications [79,80]. OCP hybridized with dicarboxylate ions is called octacalcium phosphate-carboxylate (OCPC), which is summarized in Table 1. Although there have been many reports on dicarboxylate ions, the hybrid conditions, mechanisms and the possibility of hybridizing the organic ions into the nanospace within the hydrated layer of OCP have not been clarified in detail. 

The dicarboxylate ion that has the shortest carbon chain hybridized with OCP is malonate ion [80] (R = CH_2_), which is a linear saturated dicarboxylate ion. Among the linear saturated dicarboxylate ions, the succinate ion (R = (CH_2_)_2_) with a carbon chain that is one carbon longer than the malonate ion is relatively easy to hybridize compared to other dicarboxylate ions, and the substitution rate between hydrogen phosphate and succinate ion is high. The OCP hybridized with succinate ion has been evaluated by X-ray diffraction (XRD), infra-red (IR) spectroscopy, thermal stability, nuclear magnetic resonance (NMR) spectroscopy and theoretical analysis, and it has been synthesized under various conditions [49,80,81,82,83]. The *d*_100_ values of OCPs hybridized with linear saturated dicarboxylate ions increased linearly with the length of the carbon chain (R = (CH_2_)_n_ *n* = 1–6) from 1.96 to 2.60 nm. For example, the *d*_100_ value of OCP hybridized with adipate ion (R = (CH_2_)_4_) was 2.37 nm. This relationship indicated that the hybridization form in the hydrated layer was almost the same in the OCP hybridized with linear saturated dicarboxylate ions, even if the length of the carbon chain varied. The longest linear saturated dicarboxylate ion that can be hybridized with OCP is the sebacate ion (R = (CH_2_)_8_). The linear saturated dicarboxylate ions with carbon chains longer than sebacate ions have not been reported because of low solubility in water and difficulties in ionization [79,80].

The dicarboxylate ions with side chains have also been investigated, and dicarboxylate ions with succinate ion skeleton have been compared and evaluated. For example, the *d*_100_ value of OCP hybridized with 2-methylsuccinate ion (R = CH(CH_3_)CH_2_) extended to 2.04 nm [84,85]. In the case of 2-methylsuccinate ion, there was a difference between the optical isomers in the hybridization with OCP. Specifically, there was no difference in the XRD diffraction position of OCP synthesized in the presence of the R-form of 2-methylsuccinate ion due to the 100 plane compared to the normal OCP, indicating that the R-form of 2-methylsuccinate ion did not hybridize. By contrast, OCP synthesized in the presence of S-form 2-methylsuccinate ion exhibited different XRD intensities, a reduced Ca/P molar ratio and an increased carbon content compared to normal OCP, suggesting the partial substitution of S-form 2-methylsuccinate ion. The results indicated that OCP discriminated the chirality of the dicarboxylate ions, leading to potential applications as catalysts and adsorbents [89]. In the case of the 2-mercaptosuccinate ion (R = CH_2_CH(SH)) with a mercapto group (-SH) on the side chain of the succinate ion, the *d*_100_ value increased continuously from 1.87 nm to 2.12 nm with increasing 2-mercapto-succinate ion modification concentration [86]. According to these results, the presence of the side chains may alter the state of hydrogen bonding between water molecules and hydrogen phosphate ions within the hydrated layer of OCP, which affect the crystal structure, adsorption ability and thermal stability.

The unsaturated dicarboxylate ions can also be hybridized with OCP. The unsaturated dicarboxylate ion hybridized with OCP is the fumarate ion (R = CH = CH, *trans*) with the shortest carbon chain, and a *d*_100_ value is 2.15 nm [80]. However, the malate ion (R = CH = CH, *cis*), which is a geometric isomer with the same chemical formula as the fumarate ion, does not hybridize with OCP. The *d*_100_ values of OCP hybridized with unsaturated dicarboxylate ions are not consistent with those of OCP hybridized with saturated dicarboxylate ions in the same carbon chain length.

The aromatic dicarboxylate ions can be hybridized with OCP. A comparison was carried out between three benzene dicarboxylate ions (R = C_6_H_4_); phthalate (ortho isomer), isophthalate (meta isomer) and terephthalate ion (para isomer) that had dicarboxylate ions bound to different positions on the benzene ring, and only isophthalate ion (meta isomer) was hybridized with OCP [87]. Despite having two carboxylate ions in the para position, which were not hybridized with OCP, in the case of benzene dicarboxylate ion, 2,2′-bipyridine-5,5′-dicarboxylate (bpdc) ion (R = (C_5_H_3_N)_2_) with two pyridine ring was successfully hybridized with OCP, and the luminescence due to the pyridine ring was observed [88]. Accordingly, the molecular structure of the dicarboxylate ion is assumed to influence the hybridization with OCP. Because water molecules and hydrogen phosphate ions in the OCP hydrated layer form the complexed hydrogen bonds, dicarboxylate ions with hydrogen bondable sites in the molecular structure are likely to hybridize with OCP. Moreover, the further functionalization of the OCP is possible by hybridizing dicarboxylate ions with specific functions (e.g., photofunction) into the hydrated layer of the OCP.

#### 2.1.4. Photofunctionalization Based on the Biomimetic Hybridization

Although there are few reports on the photofunctionalization of OCP, luminescent dicarboxylate ions have been hybridized into the hydrated layer by the substitution property of hydrogen phosphate ions with dicarboxylate ions. Bpdc ion acting as a ligand to form a luminescent metal complex is an organic ion and has weak luminescence. The hybrid of OCP and bpdc ion exhibited the blue luminescence, which is similar to that of bpdc ion in the solution state, with higher efficiency than neat bpdc [88]. Moreover, the pyromellitate ion, which is not a dicarboxylate but a tetracarboxylate ion with an aromatic ring, can be hybridized into the hydrated layer of OCP, and the hybrid of OCP and pyromellitate ion has a more intense blue luminescence than pyromellitic acid alone [90]. These are the only two examples of the utilization of organic ions to impart the luminescent properties to OCP.

### 2.2. Hydroxyapatite

#### 2.2.1. Features

**Figure 5:**
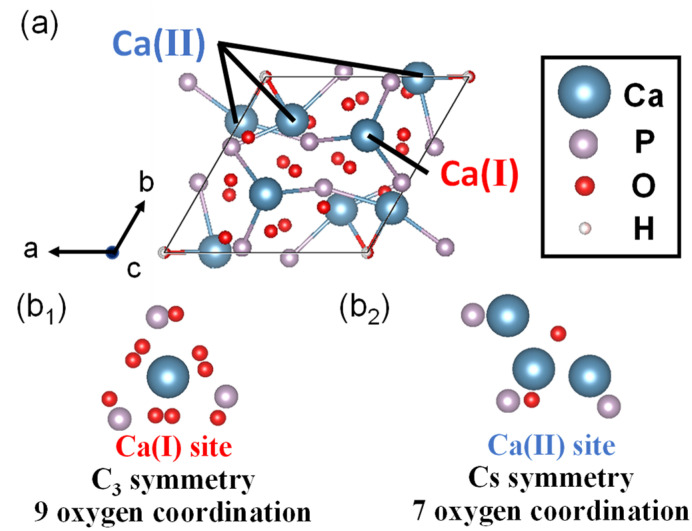
(**a**) Crystal structure of HA and (**b_1_**,**b_2_**) illustration and symmetry of two Ca sites.

HA (Ca_10_ (PO_4_)_6_ (OH)_2_) is a type of CP, has a Ca/P molar ratio of 1.67 and density of 3.16 g∙cm^−3^ and belongs to the hexagonal crystal system and space group of P6_3_/m. The lattice constants of HA are *a* = *b* = 9.41, *c* = 6.88 Å, *γ* = 120° [47]. In the HA crystal structure (Figure 5a), the four Ca sites with C_3_ symmetry, called columnar Ca (Ca (I) sites, Figure 5(b_1_)), are aligned parallel to the c-axis, and the six Ca sites with C_S_ symmetry, called screw axis Ca (Ca (II) sites, Figure 5(b_2_)), surround the OH groups present in the four corners of the HA unit cell [91]. HA is an inorganic component found in biological tissues, such as teeth and bones, and is a stable CP with high biocompatibility. The solubility (−logKsp) of HA at 25 °C is 116.8 and at 37 °C—117.2, and HA is stable at a pH value of 9.5 to 12 in aqueous solution at 25 °C [50,51]. HA is used as an artificial bone because of its similarity to the bone composition in vivo [92,93,94]. HA is also used as an adsorbent due to the adsorption properties and interactions with bio-related substances, such as nucleic acids [95,96], proteins [97,98] and pollutants [99,100].

#### 2.2.2. In Vivo and In Vitro Formation Processes

**Figure 6:**
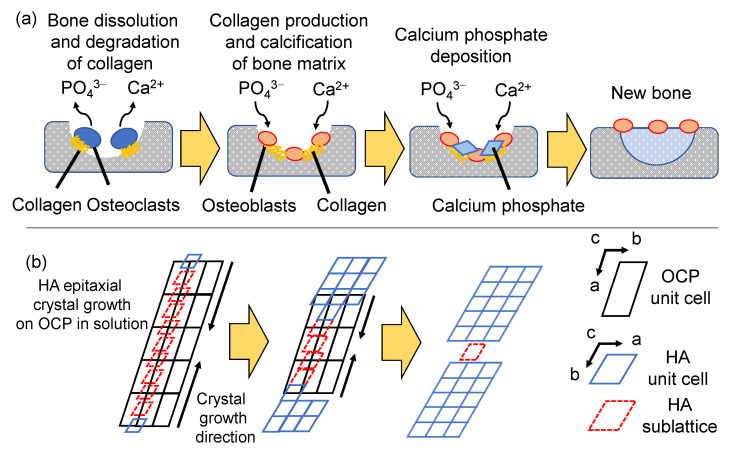
Illustration of (**a**) bone regeneration mechanism in vivo and (**b**) formation mechanism of HA on OCP in vitro.

Since HA is a major inorganic component in bone, HA is produced while taking in the surrounding ions (e.g., Na^+^, Mg^2+^, K^+^) [101] through an osteogenic (bone formation) process in vivo. Although some aspects of the osteogenic process remain unclarified, the process involves the following steps. The process of HA formation in vivo is illustrated by using the bone regeneration mechanism as an example shown in Figure 6a. The mesenchymal stem cells proliferate and aggregate to form the basis of cartilage and further proliferate and secrete substances to become the cartilage tissues themselves. Part of the cells differentiates to chondrocytes, and chondrocytes secrete the type X collagen and proteins. The collagen deposits calcium in the cartilage tissue, and proteins promote the blood vessel formation. Eventually, the chondrocytes lead to apoptosis with the deposition of CPs around the chondrocytes. Subsequently, osteoblasts gather in the cartilage tissue through the blood vessels and secrete and accumulate uncalcified matrix (osteoid). When the thickness of the osteoid reaches approximately 10 μm, it calcifies from the tip. The mechanism of calcification is not completely clear. However, in terms of ionic product and HA solubility, CPs, which are more soluble than HA, appear to precipitate in osteoid and then convert to HA to form bone. Experimentally, the precursors of HA are amorphous CP (ACP) and/or OCP. The crystal structure of OCP (sublattice of HA) is recognized as a nucleation site for HA, and epitaxial growth of HA proceeds on the OCP crystal (Figure 6b) [71]. There are two main methods of synthesizing HA: dry and wet processes. The wet process is significant in biomaterials because water is always present in living organisms and is essential for the synthesis of HA by mimicking the reactions in vivo while interacting with organic substances. There are various wet processes for HA synthesis—mainly sol-gel, chemical precipitation and hydrothermal synthesis, or a combination of these methods.

As the examples of the sol-gel method the HA particles with a size of 200 nm can be obtained by hydrolysis of (C_2_H_5_)_3_PO_4_ in ethanol containing water (Equation (4)), followed by reaction with Ca(NO_3_)_2_ and calcination at 800 °C [102]. The HA nanoparticle with a size of 10–80 nm can be obtained by the reaction of P_2_O_5_ ethanol solution and Ca(NO_3_)_2_ ethanol solution for 4–72 h, subsequent calcination at 700 °C, and the particle size increases with increasing reaction time [103].

P(OEt)_3−x_(OH)_x_ + Ca^2+^ + NO_3_^−^ → (NO_3_)(OH)–Ca–O–PHO(OEt)_3−x_ + H^+^ + C_2_H_5_OH + H_2_O (4)

The following equation (Equation (5)) is common for HA synthesis using water-soluble calcium and phosphate salts. In the case of HA synthesis via such an ionic reaction, the ACP is produced as a precursor [104]. Although the details of the ACP structure are still unknown, ACP is considered to have a structure similar to that of the Ca(I) site in the unit lattice of HA and to be composed of spherical clusters with a Ca/P molar ratio of 1.5 (Ca_3_(PO_4_)_2_) [105].

10Ca^2+^ + 6PO_4_^3−^ + 2OH^−^ → Ca_10_(PO_4_)_6_(OH)_2_ (5)

Hydrothermal synthesis is a method of synthesizing highly crystalline HA by reacting an aqueous solution containing calcium and phosphate sources in an autoclave with a reaction temperature of 90–400 °C [106]. HA particles with different morphologies (e.g., belts (width: 30 nm and length: 850 nm) and nanorods (width: 12 nm and length: 40 nm)) are synthesized depending on the solvent type, initial concentrations and pH [107]. Various HA morphologies can also be obtained by the coexistence of organic molecules in the reaction system. HA nanorod with a width of 10 nm and a length of over 100 μm can be synthesized by hydrothermal synthesis in an aqueous solution containing oleic acid and methanol [108]. Chemical precipitation is the most widely applied method for the synthesis of HA and is characterized by low temperature reactions, low crystallinity and the production of various HA morphologies depending on the experimental conditions [109]. In the synthesis of HA by the reaction of Ca(OH)_2_ and H_3_PO_4_, with the increase in temperature from 40 to 100 °C, the synthesized HA particles vary from needle-like crystals (width: 25 nm and length: 200 nm) to spherical particles (diameter: approximately 50 nm), and the trend is reversed for reactions of water-soluble salts. The difference in morphology is related to the growth rate; faster rates lead to the generation of needle-like crystals, and slower rates lead to the generation of spherical particles [110]. Therefore, in the synthesis of HA using water-soluble salts, larger HA particles with higher aspect ratios are obtained at higher temperatures [111]. A variety of functional HA particles have been synthesized based on these synthetic methods.

#### 2.2.3. Hybridization with Organic Molecules in the Formation Process

As described in Section 2.2.2, HA is the main inorganic component of bone, while collagen is the main organic component of bone [112]. In the application of bone filling materials, many collagen and HA hybrids have been reported to mimic the bone tissue [113,114,115]. The collagen and HA are hybridized by forming covalent bonds between the COO^−^ ion of the collagen and the Ca^2+^ ion on the HA surface. By using these covalent bonds, the hybrids of HA and gelatin [116,117], which are similar to the structure of collagen, chitin [118,119], chitosan [120,121], polycarboxylate [122], have been synthesized for bone filling material applications. In addition to the hybridization with organic molecules via the interaction between surface Ca^2+^ ion on HA and COO^−^ ion of organic molecules, the hybridization of HA and cationic organic molecules is expected via the interaction between surface phosphate ions and positively charged functional groups.

#### 2.2.4. Photofunctionalization Based on the Biomimetic Hybridization

Cyanine dyes—a type of fluorescent dye that is used for fluorescent staining for nucleic acids and proteins—have various excitation and emission wavelengths depending on the chemical structure of the dye, such as Cy3 (excitation wavelength: 555 nm, emission wavelength: 570 nm) and Cy5 (excitation wavelength: 646 nm, emission wavelength: 662 nm). The Cy3 and CP hybrid particles had superior luminescence properties compared to Cy3 alone, with approximately 10 times stronger luminescent intensity and 4 times higher internal quantum efficiency [123]. Fluorescein isothiocyanate (FITC), a fluorescent dye, can be directly adsorbed onto HA particles and used for gastric cancer cellular imaging by modifying the antibodies [124]. These fluorescent dyes have a carboxy group, and the interaction between Ca^2+^ and COO^−^ ions is assumed to lead to efficient adsorption of fluorescent dyes. On the other hand, HA is used as an adsorbent due to its low environmental impact and ability to adsorb various inorganic and organic substances. In addition to the above-mentioned interaction between Ca^2+^ and anion groups, the interaction between cations with phosphate ions, hydrogen bonding and ion exchange have been proposed as adsorption mechanisms [100,125,126]. In other words, if photofunctional dyes coexist during synthesis, they can be hybridized via these interactions.

## 3. Utilization of Europium(III) (Eu^3+^) Ion Doped in CPs

### 3.1. Photophysical Properties of Eu^3+^ Ion

One of the photofunctionalization methods of CPs is rare earth ion (e.g., Eu^3+^, Gd^3+^, Tb^3+^, Tm^3+^, Yb^3+^) doping [127,128,129]. Rare earth ions exhibit luminescence when their symmetry is broken (e.g., immobilization in the solid phase and coordination of molecules). Among the rare earth ions, Eu^3+^ ion is less toxic and has a red emission in visible light with relatively higher tissue permeability, which is suitable for visual imaging in biological tissue. Accordingly, this section describes the characteristics of Eu^3+^ ion and their hybrid systems in CPs and prospects.

Eu^3+^ ion has 60 electrons—54 electrons in the same closed shell structure as the xenon atom and 6 electrons in the 4f shell. The electronic structure of the Eu^3+^ ion can be represented as [Xe] 4f^6^ or 4f^6^ for short. The possible electron configurations of the Eu^3+^ ion are 3003, and Hund’s rule applies in the ground state. In general, these electron configurations can be expressed using the Russell–Saunders term symbol (Equation (6)).

(6) L 2S+1J

where *S* is the total spin quantum number, *J* is the total angular momentum quantum number, and *L* is the symbol assigned to the total orbital angular momentum quantum number. The symbols assigned to *L* are S (*L* = 0), P (*L* = 1), D (*L* = 2), F (*L* = 3), G (*L* = 4), H (*L* = 5), I (*L* = 6), K (*L* = 7), L (*L* = 8), M (*L* = 9), …, and after the F symbol, the letters are arranged in alphabetical order, excluding *J*. In the Eu^3+^ ion (4f^6^) of the ground state, the 2*S* + 1 is 7, and *L* is 3 and is denoted by ^7^F*_J_* (*J* = 0).

**Figure 7:**
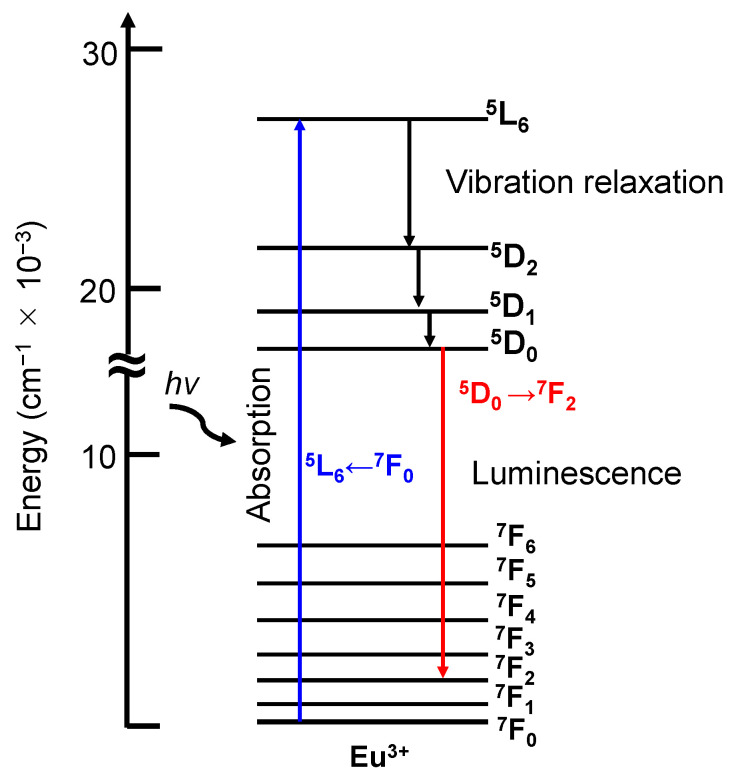
Partial energy diagram of Eu^3+^ as an example of complex. As an example, the blue arrow is ^5^L_6_ ← ^7^F_0_ transition meaning excitation and the red arrow is ^5^D_0_ → ^7^F_2_ transition meaning luminescence.

Some Eu^3+^ ion complexes and compounds exhibit a strong luminescence from the ^5^D_0_ excited state to the *J* level of the ground state ^7^F (i.e., derived from the ^5^D_0_ → ^7^F*_J_* transition (*J* = 0–6)) (Figure 7). Thus, they are widely used in a fluorescent tube as red fluorophores (e.g., Y_2_O_3_:Eu^3+^) and are also applied as luminescence probes in the biochemical and biomedical field. Since the ^5^D_0_ → ^7^F_2_ and ^5^D_0_ → ^7^F_4_ transitions are sensitive to the surrounding coordination environment of Eu^3+^ ion, their peak position and relative intensities in the absorption and luminescence spectra provide the information on the local environment (point group symmetry and coordination structure) of the Eu^3+^ ion [29].

The luminescence of Eu^3+^ ion can be divided into two main types: magnetic dipole transitions and electric dipole transitions. The ^5^D_0_ → ^7^F_1_ transition, which is a magnetic dipole transition, is observed between 585 and 600 nm. Due to the property of the magnetic dipole transition, the total integrated intensity is almost independent on the Eu^3+^ ion symmetry. Accordingly, the integrated intensity of this transition can be used to calibrate the luminescent intensity to compare the different systems. This transition is strongly observed when the Eu^3+^ ion is present at the high symmetry sites.

The electric dipole transitions are usually forbidden, but symmetry breaking due to the ligand field allows these transitions. Strong crystal field effects are required to obtain strong luminescence. In the Eu^3+^ ion, the ^5^D_0_ → ^7^F_0_ (570–585 nm), ^5^D_0_ → ^7^F_2_ (610–630 nm), ^5^D_0_ → ^7^F_3_ (640–660 nm) and ^5^D_0_ → ^7^F_4_ (680–710 nm) transitions are electric dipole transitions. The relative intensity of the ^5^D_0_ → ^7^F_0_ transition is very weak. When this transition is observed, the coordination environment of the Eu^3+^ ion has some specific symmetries (e.g., C_n_, C_nv_, C_s_ symmetry). These properties provide information about the substitution sites of the Eu^3+^ ion. The ^5^D_0_ → ^7^F_2_ transition is the most sensitive transition of the Eu^3+^ ion to the coordination environment. From this property, the intensity of this transition is used as an index for the Eu^3+^ ion site asymmetry. For example, the symmetry of the Eu^3+^ ion site can be discussed using the ratio of the integrated intensity of this transition to that of the ^5^D_0_ → ^7^F_1_ transition. The ratio is relatively higher at the lower symmetry of the Eu^3+^ ion site than that at the higher symmetry site [130]. The ^5^D_0_ → ^7^F_3_ transition is rarely used in applications because this transition only has a very weak luminescence. The luminescence intensity of the ^5^D_0_ → ^7^F_4_ transition is relatively high and depends on the Eu^3+^ ion coordination environment. However, the luminescent wavelength of this transition is not sensitive to the commonly used detectors, which makes it difficult to compare with other systems.

Doping with Eu^3+^ ions provides not only the base material with luminescent properties but also information on the substitution sites and coordination environment by comparing the luminescence intensity ratios. Moreover, it is important to reduce the symmetry to increase the fluorescence intensity.

Due to the luminescent properties, Eu^3+^ ion has been applied in cancer cell imaging in various forms (e.g., complexes [131], complex hybrid nanoparticles [132] and ion-doped nanoparticles [133,134,135]). Moreover, theranostic nanomaterials have been proposed by combining drug molecules with Eu^3+^ -doped nanoparticles [136,137]. On the basis of the relationship between the luminescence intensity of Eu^3+^ ion and the drug loading amount, it is expected that not only imaging but also the released amount of the drug molecule can be estimated from luminescence intensity.

### 3.2. Doping System in CPs

As described earlier in Section 2.2, the CPs have the ability to adsorb and substitute a variety of different ions in the surrounding environment when the CPs are formed in vivo. This property has been widely used in research to functionalize CPs by substituting ions with various properties. Among the CPs, the HA has been widely studied because of its easy synthesis, high substitution capacity and high biosafety. For example, various ions have been substituted, such as transition metal ions (e.g., Ni^2+^ [138] and Co^2+^ [139]) in catalysis, antibacterial metal ions (e.g., Ag^+^ [140], Zn^2+^ [141] and Mg^2+^ [142]) in medical applications, biological ions (e.g., Na^+^, K^+^ [101] and Fe^3+^ [143]) and ions that promote bone formation and regeneration (Mg^2+^ [144], Sr^2+^ [145] and Zn^2+^ [144]). Moreover, luminescent ions (e.g., Eu^3+^ [146,147,148] and Tb^3+^ [148,149] ions) can be doped into the HA crystal structure. These luminescent ions are expected to be used as a probe for changes in crystal phase [150]. The luminescence spectrum of Eu^3+^-doped HA differs depending on the substitution site. Specifically, the luminescence of Eu^3+^ substituted at the Ca (I) site with oxygen-9 coordination and C3 symmetry was observed around 578 nm, while that substituted at the Ca (II) site with oxygen-7 coordination and Cs symmetry was observed around 574 nm [130]. The combination of HA with biosafety and luminescence derived from rare earth ion has been proven to be useful as both bioimaging material and drug carrier [130,151].

OCP could also incorporate a variety of ions at the Ca site in the crystal structure owing to the similarity in crystal structure with HA [152]. In fact, the substitution of various ions into the OCP has been considered for the functionalization of OCP (e.g., Mg^2+^ [153], Sr^2+^ [154,155], Zn^2+^ [153,156], Fe^3+^ [155] and Ag^+^ [157,158]) (Table 2). Metal ion substitution is most likely to occur when the ionic radii of the two species are similar; the Ca^2+^ ions have the ionic radii of 1.06 Å (7-coordination), 1.12 Å (8-coordination) and 1.18 Å (9-coordination). There are eight Ca sites in the OCP, and the Ca3 and Ca8 sites tend to be substituted by relatively large cations, while the Ca6 site tends to be substituted by small cations [154]. 

Magnesium ions (Mg^2+^, ionic radius: 0.89 Å (8-coordinated) and 0.65 Å (5-coordinated)) activate osteoblasts and osteoclasts in vivo. On the other hand, Mg^2+^ ion competes with Ca^2+^ ion adsorption, leading to the inhibition of HA formation and enamel crystal growth. As the ionic radius of Mg^2+^ ion is smaller than that of Ca^2+^ ion, the lattice parameter tends to decrease when Ca^2+^ and Mg^2+^ ions are substituted, and the Ca6 site is a favorable substitution site [153]. The conversion of OCP to HA is inhibited at Mg^2+^ ion concentrations in the solution (Ca/(Ca + Mg)) above 2 at%, with a maximum Mg^2+^ ion solid solution of approximately 10 at% [69,159]. Mg^2+^ ion has an inhibitory effect on crystallization. The presence of more than 20 at% in solution inhibits the formation of OCP, and Mg^2+^ ion doping reduces the thermal stability of the layer structure.

Strontium ions (Sr^2+^, ionic radius: 1.21 Å (7-coordinated), 1.26 Å (8-coordinated) and 1.31 Å (9-coordinated)) promote the differentiation of pre-osteoblasts into osteoblasts, activate their function and inhibit the differentiation and activity of osteoclasts, promoting bone formation. Sr^2+^ ion doping into OCP has been conducted to improve the osteogenic activity, and up to 7 at% was introduced in OCP single phase. The structure of OCP was maintained up to a Ca/(Ca + Sr) concentration of 7 at%, and the substitution sites are considered to be Ca3, Ca4 and Ca8 sites [155]. Sr^2+^-doped OCP tends to have an increased lattice parameter, and large amounts of Sr^2+^ ion inhibit the nuclear growth of OCP [69]. The zinc ion (Zn^2+^, ionic radius: 0.68 Å (5-coordinated), 0.74 Å (6-coordinated) and 0.90 Å (8-coordinated)) is an essential trace element in living organisms. Zn^2+^ ion promotes osteoblast differentiation and inhibits osteoclast differentiation leading to bone formation [160,161]. The conversion of Zn^2+^-doped OCP to HA was suppressed, and the large amount of Zn^2+^ ion reduced the crystallinity and promoted the formation of ACP [156]. Theoretical calculation on Zn^2+^-doped OCP has been carried out, which indicated that the Ca6 site is favorable for substitution [153]. Iron ions (Fe^3+^, ionic radius: 0.58 Å (5-coordinated), 0.65 Å (6-coordinated) and 0.78 Å (8-coordinated)) are essential trace elements in living organisms and are mainly found in bones and teeth, where they are involved in cell metabolism and proliferation [155,162]. Since the ionic radius of the Fe^3+^ ion is smaller than that of the Ca^2+^ ion, the lattice constant of OCP should decrease in the case of Fe^3+^ ion doping into OCP. However, the constant increases, suggesting that Fe^3+^ is substituted at the Ca8 site in the form of Fe (OH)^2+^ [155]. 

### 3.3. Bioimaging Application with OCP

The aforementioned biosafety of CPs, COO^−^ mediated hybridization with organic molecules and the incorporation of different ions have led to a number of bioimaging applications. Some examples are given below. The co-doping of Eu^3+^ and Gd^3+^ ions in CP showed excellent biocompatibility for cellular experiments, high biodegradability (65% decomposition in 72 h) and in vivo imaging [163]. Silicate-substituted HA doped with Bi^3+^ ion enhanced the cell activation and had osteogenic and luminescence properties for bone regeneration and bioimaging [164]. Furthermore, by doping Gd^3+^ and Eu^3+^ ions into CPs and loading ibuprofen as a model molecule for anticancer drugs, CP was used as a multifunctional drug carrier for long-term sustained release of drug molecules, visible light imaging by Eu^3+^ ion and nuclear magnetic resonance imaging by Gd^3+^ ion [165]. While there are many examples of using HA for bioimaging applications, the application of OCP has not been investigated widely. OCP can hybridize with organic ions within the OCP crystal structure, which is not found in HA. If the OCP can be photofunctionalized by ion doping in combination with organic modification of the interlayer, it is possible to design multifunctional OCP with biosafety.

## 4. Utilization of Methylene Blue Adsorbed on HA

### 4.1. Photophysical and Photochemical Properties of Methylene Blue

**Figure 8:**
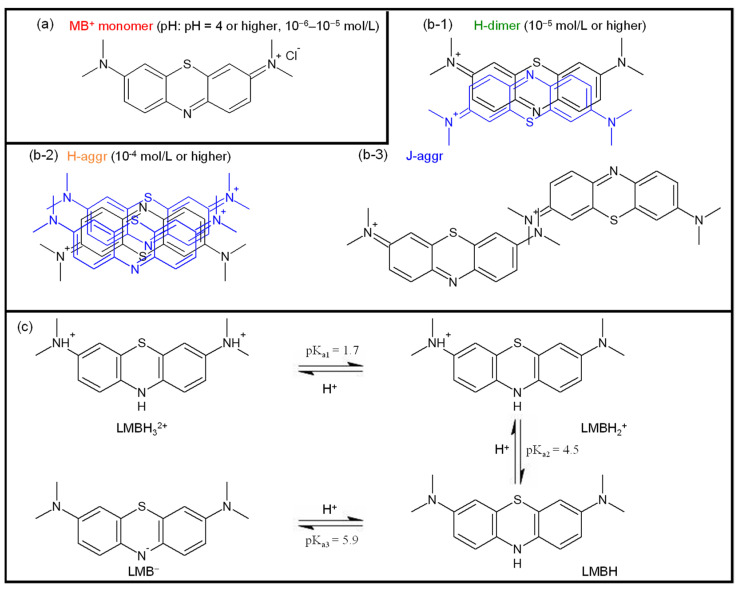
Chemical structures in aqueous solution of (**a**) MB^+^ monomer, which generates ^1^O_2_, (**b-1**–**b-3**) MB^+^ aggregation states at the different concentrations and (**c**) acid-based equilibria of LMB in water.

Figure 8 shows the molecular structures of methylene blue (MB^+^) and their aggregates and derivatives. MB^+^ is a cationic dye that has absorption at 293 nm (π-π*) and 665 nm (n-π*) in monomer (Figure 8a) in aqueous solution [166]. With increasing concentration, MB^+^ forms face-to-face aggregations. The maximum absorption wavelengths (λ_max_) are 605 nm for the dimer (H-dimer, Figure 8(b-1)) and 575 nm for the trimer or higher aggregate (H-aggr, Figure 8(b-2)) [166]. The equilibrium constant (K_1_) for the dimerization reaction (2MB^+^ → H-dimer) is reported to be 2–10 × 10^3^ M^−1^, and that (K_2_) for the trimerization reaction (H-dimer + MB → H-aggr) is 6 × 10^6^ M^−2^ [166]. The formation of head-to-tail aggregates (J-aggr, Figure 8(b-3)) has also been suggested when adsorbed on clay minerals, such as montmorillonite [167]. Moreover, the MB^+^ also forms derivatives depending on the pH of the solution; under strong acid conditions at pH < 2, protonated MB^+^ (MBH, λ_max_ = 760 nm) is formed, while at pH 2–7, leucomethylene blue (LMB, λ_max_ = 314 nm, Figure 8c), a reduced form of MB^+^, is formed. The MB^+^ is expected to be used for cancer imaging applications because of their luminescence in the red-color light range (686 nm) [168], which is highly tissue permeable. For example, 24 breast cancer patients before surgery were given 1.0 mg/kg MB^+^ by passive diffusion into the tumor with intravenous injection. After the tumor sites were identified by MB^+^ luminescence and excised during surgery, the excised part was observed using a fluorescence microscope to confirm the presence of the tumor. In 20 out of 24 patients, the presence of a tumor was confirmed at the excised part, which offers the possibility of real-time breast cancer detection [169].

**Figure 9:**
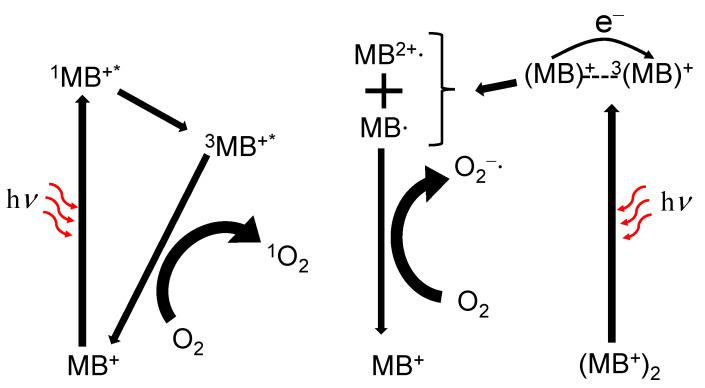
Methylene blue photochemical reaction routes where MB^+^, ^1^MB^+*^, ^3^MB^+*^ are methylene blue ground state, singlet and triplet excited states, respectively; MB∙ and MB^2+^∙ are methylene blue semi-reduced and semi-oxidized radicals, respectively.

In addition to luminescent properties, MB^+^ has photosensitizing properties and two pathways for the photosensitization reaction: type I by electron transfer and type II by energy transfer (Figure 9). MB^+^ produces the ^1^O_2_ by type II in monomer state and superoxide ion (O_2_^−^**·**) by type I in H-dimer [33]. In MB^+^ monomer, the ground state MB^+^ transitions to the excited singlet state (^1^MB^+*^) by electronic transitions due to the visible light absorption. Then, the ^1^MB^+*^ state transitions to the excited triplet state (^3^MB^+*^) by intersystem crossing. The ^1^O_2_ is produced by energy transfer between ^3^MB^+*^ and the triplet oxygen (^3^O_2_), which is the ground state of O_2_ present in solution, with a ^1^O_2_ generation efficiency (Φ_Δ_) of approximately 0.5 [33,170]. In the excitation of H-dimer, the ground state of MB^+^ donates an electron to the ^3^MB^+*^, producing semi-reduced radical (MB·) and semi-oxidized radical (MB^2+^·), and this reaction is the deactivation process of ^3^MB^+*^ [33]. Moreover, the ^3^O_2_ is reduced by the MB· to form O_2_^−^. The MB^+^ has also been applied to PDT because the MB^+^ has high Φ_Δ_ in solution and can be excited by red-color light with high biological tissue permeability [33].

MB^+^ can be used for theranostics due to the luminescence and the ability to generate ^1^O_2_. The optical properties of MB^+^ can be improved by hybridizing with nanoparticles. Hybridization of MB^+^ via aptamers to magnetic core–gold shell nanoparticles red-shifted the absorption wavelength of MB^+^, enabling PDT for prostate cancer cells by near-infrared light in addition to imaging by luminescence [171]. Moreover, the hybridization of MB^+^ with gold nanoparticles enhanced the photodynamic activity for colon cancer cells while maintaining luminescence ability [172]. A combination of MB^+^ with substances for imaging is also possible. As examples, hybrid systems of MB^+^ with luminescent nanoparticles (e.g., GdF:Eu [173] and NaYF_4_:Yb, Er [174]) have been reported.

MB^+^ has not only excellent singlet oxygen generation and luminescence properties but also a wealth of knowledge due to its long history as a dye, and thus, it can be regarded as a probe as well as a photofunctional molecule. By using MB^+^ as a probe, it is possible to analogize what state of the objective carrier surfaces. Furthermore, the relationship among the variables, states of existence and photochemical properties can be elucidated, leading to the optimization of the photofunction.

### 4.2. Immobilization on HA

A number of MB^+^ adsorbed on clay compounds, including various montmorillonites and kaolinites, have been reported to determine the cation exchange capacity of clay compounds [175,176]. In these systems, MB^+^ is immobilized in the following four main forms: monomer, H-dimer, H-aggr and MBH [177,178]. The presence of J-aggr has also been suggested for some clay compounds [179]. The position of the MB^+^ absorption wavelength varies due to the changes in planarity caused by differences in the steric configuration (e.g., interlaminar and extralaminar of layered clays) of the dimethylamino groups at the ends of the MB^+^ molecule interacted with the clay surface [180].

HA has a high adsorption capacity and is used as an adsorbent for harmful metal ions and organic substances. The capacity of HA has been evaluated using MB^+^ as a model molecule for cationic harmful organic substances. The capacities of waste bone, waste-eggshell-derived HA, low-crystalline HA [181] and HA synthesized by microwave irradiation [182] have been evaluated for the removal of organic matter from wastewater. Hybrid adsorbents of HA with various organic substances have also been reported to increase the adsorption capacity for MB^+^ (e.g., arginine [183], xanthan gum [184], biochar [185], polystyrene sulphonic acid [186] and polyacrylic acid [187]).

### 4.3. Therapeutic Application

MB^+^ has potential to be used in PDT applications. However, there are several problems; the monomer that produces the ^1^O_2_ is unstable and easily converts to LMB or H-dimer that have no ^1^O_2_ generation ability, and MB^+^ has no selective accumulation in abnormal cells. Accordingly, it is important to ensure that the stable presence of the MB^+^ monomer and immobilization onto nanomaterials, such as HA with biosafety, are a promising way to solve the issues by the stabilization of MB^+^.

Therefore, the hybrids of HA and MB^+^ are developed for PDT applications, taking advantage of the ^1^O_2_ generation ability of MB^+^, biosafety and adsorption capacity of HA. The MB^+^-loaded hybrid of polystyrene sulphonic acid and HA has the ability to kill Staphylococcus aureus by laser irradiation [186]. In an MB^+^-loaded hybrid of polyacrylic acid and HA, the hybrid has a non-cytotoxicity, and the cancer cell viability after laser irradiation clearly decreased with increasing the amount of hybrid addition [187]. These results show the potential for PDT application of the hybrid of MB^+^ and HA. Moreover, the suppression of MB^+^ reduction and attempt to control the ratio of monomer to H-dimer on the particle surface have been reported in silica-based materials with nicotinamide adenine dinucleotide phosphate [188]. Moreover, the use of substances not originally present in the organism should be kept to a minimum to prevent unexpected side effects. However, no examples have been reported on direct immobilization of MB^+^ on the HA surface with controlled aggregation states.

Other examples of photofunctional organic molecules or dyes immobilized on CPs include Direct Red 23, Acid Blue 25 [189] and Reactive Yellow 4 [190], which are the model molecules for dyes in wastewater, indocyanine green as a luminescent dye [191], porphyrin [192] and hypericin [193] as a photosensitizer. Despite the fact that OCP can modify various organic molecules, there have been few reports on the immobilization of organic dyes for diagnostic or therapeutic purposes.

## 5. Conclusions and Future Perspectives

The financial and physical burden remains a problem in the diagnosis and treatment of cancer. Much of the burden is due to the poor accumulation of diagnostic and therapeutic drugs in cancer tissue, and thus, improving their accumulation can reduce the burden. For this reason, attention is focused on the nanoparticulation of drugs by carriers and theranostics, which combine diagnosis and treatment in a single system. The accumulation by the EPR effect and the modification of the particle surface can provide further functionalization and specificity. Various materials have been proposed as potential carriers. The CPs are expected to be used as a base material for theranostics due to their biosafety, biodegradability and versatility in functionalization. To realize theranostic applications, CPs need to be functionalized with photofunctional species and drug molecules through controlled interaction with Ca^2+^ or PO_4_^3−^ on the CP surface. It is, therefore, of great importance to fully understand the interaction to meet all the requirements for theranostics. Moreover, their interactions are combined to form highly functional inorganic–organic hybrids in vivo. Accordingly, imitating the surface interactions that occur in vivo may be the key to controlling the hybridization of CPs with photofunctional heterogeneous materials. In chemotherapy and PDT, the development of nanostructured CPs with a nanosize and narrow size distribution is expected for efficient immobilization of drug molecules without side effects and in the development of photofunctional CPs. Efforts are in progress to improve luminescent efficiency and intensity. Furthermore, in chemotherapy, the controlled release behavior is also necessary to control the drug concentration in the body. For applications in a wide range of diseases (mainly cancer), modification with polymer (e.g., polyethylene glycol) is necessary for blood retention, while the modification with antibodies or other agents is necessary for targeting to abnormal cells. Therefore, various knowledge is required, such as polymer chemistry, biology and medicine, as well as inorganic materials. Well-designed photofunctional CPs produced with these technologies will contribute to the biomedical field as highly functional and multifunctional theranostic nanomaterials.

## Figures and Tables

**Table 1:** Interlayer distances of OCPC hybridized with various dicarboxylate ions.

Dicarboxylate Ions	Interlayer Distance (*d*_100_)	Reference
Malonate	2.01 nm	[80]
Succinate	2.14 nm	[49,80,81,82,83]
Adipate	2.37 nm	[49,80]
2-Methylsuccinate	2.04 nm	[84,85]
2-Mercaptosuccinate	2.12 nm	[86]
Fumarate	2.15 nm	[80]
1,3-Benzenedicarboxylate	2.30 nm	[87]
2,2′-Bipyridine-5,5′-dicarboxylate	2.49 nm	[88]

**Table 2:** Effect on OCP properties and in vivo role of metal ions incorporated into synthetic OCP for biomedical fields.

Substituted Ions	Ion Radius (Å) (8-Coordination)	Effect on OCP Property	In Vivo *Role*	Reference
Mg^2+^	0.89	Inhibition of crystallization Decrease in thermal stability Decrease in crystal size	Influence on osteoblasts and osteoclasts activity	[153]
Sr^2+^	1.26	Inhibition of crystallization Decrease in thermal stability Increase in crystal size	Presence in the bone, especially at the regions of high metabolic turnover	[154,155]
Zn^2+^	0.90	Defect generation Decrease in hydrolysis rate Increase in amorphous phase generation	Stimulation of osteoblast activity (promote bone formation)	[153,156]
Fe^3+^ FeOH^2+^ Fe(OH)_2_^+^	0.78 - -	Lattice expansion	Essential elements for the cell	[155]
Ag^+^	1.28	Facilitation of OCP generation	Antibacterial activity	[157,158]
